# Supplementary material for: Hydrophilic Sulfonate Covalent Organic Frameworks for Serum Glycopeptide Profiling
Source: Int J Mol Sci. 2025 Feb 24;26(5):1957. doi: 10.3390/ijms26051957 (PMC11900406; doi:10.3390/ijms26051957)
Supplement: Supplementary file 1 [file ijms-26-01957-s001.zip › ijms-3439244-SI.pdf]

Supporting Information

Hydrophilic Sulfonate Covalent-Organic Frameworks for Serum Glycopeptide Profiling

Shishu Yang, Yuheng Jiang, Shijie Jiang, Lihong Liu, Si Liu, Hua Zhang and Zhiyuan Gu

Table of Contents

Section 1. Methods **S2**
  Characterization **S2**
  Calculation Method **S2**
  In Situ Isotope Dimethyl Labeling of Glycopeptides **S3**
  Sugar Cutting Treatment **S3**
  Liquid Chromatography-Mass Spectrometry **S3**
  Database Searching **S4**
Section 2. Sample Preparation **S4**
  Materials **S4**
  Synthesis of TpPa COF **S5**
  Synthesis of SCOF-1 .... **S5**
  Synthesis of SCOF-3 .... **S6**
  Synthesis of SCOF-4 .... **S6**
  Tryptic Digestion of Proteins **S7**
Section 3. Supporting Figures **S9**
Section 4. Supporting Tables **S29**
Section 5. Supporting Reference **S31**

Section 1. Methods

Characterization

Powder X-ray diffraction (XRD) patterns were conducted at a scan rate of 1° min^{-1} over a range of 2-50° (2θ) on an X′ Pert3 Powder (PANalytical B.V.) with Cu-Kα source (λ=1.5418 Å). Scanning electron microscopy (SEM) measurement was performed on a FEI Nova NaoSEM 450. Transmission electron microscopy (TEM) images and the highangle annular dark-field scanning TEM (HAADF-STEM) were obtained by the Thermo Scienific-Talos F200i S/TEM with an operating voltage of 200 kV. A MicrotracBEL BELSORP MINI X was used to analysis the N_2 adsorption/desorption isotherms at 77 K. The water contact angles were measured by a contact angle analyzer (Dataphysics-OCA20). Thermogravimetric analysis (TGA) was carried out on a TA SDT Q600. Fourier-Transform Infrared Spectroscopy (FT-IR) was measured by IRTracer 100 Shimadzu, Japan. The zeta potential was measured by Malvern-Zetasizer Nano S90. The X-ray photoelectron spectra (XPS) was analyzed by a Thermo Scienific ESCALAB 250Xi.

Calculation Method

All density functional theory (DFT) calculations were carried out in the CP2K code.[S1] All calculations employed a mixed Gaussian and planewave basis sets. Core electrons were represented with norm-conserving Goedecker-Teter-Hutter pseudopotentials,[S2] and the valence electron wavefunction was expanded in a double-zeta basis set with polarization functions[S3] along with an auxiliary plane wave basis set with an energy cutoff of 400 Ry. The generalized gradient approximation exchange-correlation functional of Perdew, Burke, and Enzerhof (PBE) was used. Each configuration was optimized with the Broyden-Fletcher-Goldfarb-Shanno (BGFS) algorithm with SCF convergence criteria of 1.0×10^{-6} au. The van der Waals correction of Grimme's DFT-D3 model was also adopted.[S4] The Brillouin-zone integration was sampled with a Γ-centered Monkhorst-Pack mesh of 1 × 1 × 1.

The adsorption energy between the adsorbate and the substrate can be calculated

using the following equation:

$$\Delta E_{ads} = E_{adsorbate@\text{substrate}} - E_{\text{substrate}} - E_{adsorbate} \quad \text{(S1)}$$

In Eq. (S1), $E_{adsorbate@\text{substrate}}$ and $E_{\text{substrate}}$ represent the total energies of the substrate with and without the adsorption of adsorbate, respectively. $E_{adsorbate}$ is the total energy of the adsorbate. According to this equation, a negative adsorption energy corresponds to a stable adsorption structure.

In Situ Isotope Dimethyl Labeling of Glycopeptides

In situ isotope labeling was performed after glycopeptides capturing and non glycopeptides washing steps and before the elution step. At that time, the SCOF-2 with adsorbed glycopeptides were dispersed into a 200 μL CH_3COONa buffer solution (100 mM, pH ~ 5.8) and added with 8 μL of CD_2O or CH_2O and 8 μL of 600 mM $NaBH_3CN$. After being vortexed for 40 min, 10 μL of 88% formic acid (FA) was added to terminate the labeling reaction. Rinsing with 200 μL of washing buffer (90% ACN/9.9% H_2O/0.1% TFA, v/v/v) was performed to remove the excess labeling reagents. Finally, 10 μL elution buffer (30% ACN/69.9% H_2O/0.1% TFA, v/v/v) was added to the SCOF-2 and vibrated rapidly for 30 min to elute the adsorbed hydrophilic peptides. The eluent was analyzed by MALDI-TOF MS.[S5]

Sugar Cutting Treatment

The lyophilized human serums glycopeptides were resuspended to 50 mM NH_4HCO_3 (prepared with heavy oxygen water) and fully dissolved, then 6 μL PNGase F enzyme (prepared with heavy oxygen water) was added and the reaction was performed for 2 h at 37 °C.

Liquid Chromatography-Mass Spectrometry

All analyses were performed by a timsTOF HT mass spectrometer (Bruker) equipped with an CaptiveSpray source (Bruker, GER). Nanoflow reversed-phase chromatography was performed on an EASY-nLC 1200 system (Thermo Fisher Scientific). Mobile phases A and B were 0.1 vol% FA solution and 80:20:0.1 vol%

ACN:H_2O:FA, respectively. The total run was 30 min (0~28 min, 4% B; 28~29 min, 32% B; 29~30 min, 100% B; 30 min, 100% B). Capillary voltage was 1.75 kV, dry gas temperature was 180 °C, and dry gas flow rate was 3.0 L/min. The full MS scan range was set from 100 to 1700 m/z. The ion mobility range was 0.6-1.6 vs/cm^2, and the collision energy range was 20-60 eV.

Database Searching

MS/MS spectra were searched using the Spectronaut Pulsar 18.7 (Biognosys, Swiss) against the uniprot-Homo sapiens-9606-2024.2.1.fasta database. Search database specific parameters are set as follows: Fixed modifications: Carbamidomethyl (C); Variable modification: Oxidation (M), Acetyl (Protein N-term); Digestion: Trypsin/P; Precursor Qvalue cutoff: 0.01; Protein Qvalue cutoff: 0.01; Missed cleavage: 2; Quantity MS-Level: MS2.

Section 2. Sample Preparation

Materials

Triformylphloroglucinol (Tp), benzene-1,4-diamine (Pa), 2,5-diaminobenzenesulfonic acid (Pa-SO_3H), 2,5-diaminobenzene-1,4-disulfonic acid (Pa-$(SO_3H)_2$), 4,4'-diaminobiphenyl-3,3'-disulfonic acid (BD-$(SO_3H)_2$) and 1,3,5-tris(4-formylphenyl)benzene (TFPB) were obtained from Jilin Chinese Academy of Sciences - Yanshen Technology Co., Ltd., China. 3-indoleacetic acid (IAA), dithiothreitol (DTT), 1,4-dioxane, mesitylene, dimethylacetamide (DMAC), tetrahydrofuran (THF), ethanol (C_2H_5OH), dichloromethane (DCM), ammonium bicarbonate (NH_4HCO_3), acetate (CH_3COOH), sodium acetate (CH_3COONa), formaldehyde solution (CH_2O), deuterium formaldehyde (CD_2O) and formic acid (FA) were purchased from Aladdin Chemistry Co., Ltd. Acetonitrile (ACN), trifluoroacetic (TFA), trypsin (TPCK-treated), bovine serum albumin (BSA, from bovine milk), horseradish peroxidase (HRP), human serum immunoglobulin G (IgG) and PNGase F enzyme were obtained form Sigma-Aldrich (St. Louis, MO). Sodium cyanoborohydride ($NaBH_3CN$) and α-cyano-4-hydroxycinnamic acid (CHCA) were obtained from TCI (Shanghai, China). Acetone

(C_3H_6O) were obtained from Fuyu (Tianjin, China). All chemicals used were of analytical grade.

Synthesis of TpPa COF

Tp (31.5 mg, 0.15 mmol) and Pa (24 mg, 0.225 mmol) were added to a Pyrex tube containing a mesitylene/1,4-dioxane (1/1 (v/v)) mixed solvent (1.5 mL) with 3 M AcOH (0.3 mL), which was then sonicated for 10 min to get a homogeneous mixture. The reaction mixture was flash-frozen under liquid nitrogen and degassed by three freeze–pump–thaw cycles. Then, the tube was sealed and heated at 120 °C for 72 h under a static condition. The resultant precipitate was collected by filtration and washed with anhydrous THF, DCM, and acetone for three times. After drying under vacuum at 120 °C for 12 h, the TpPa COF was obtained as a red powder.[S6]

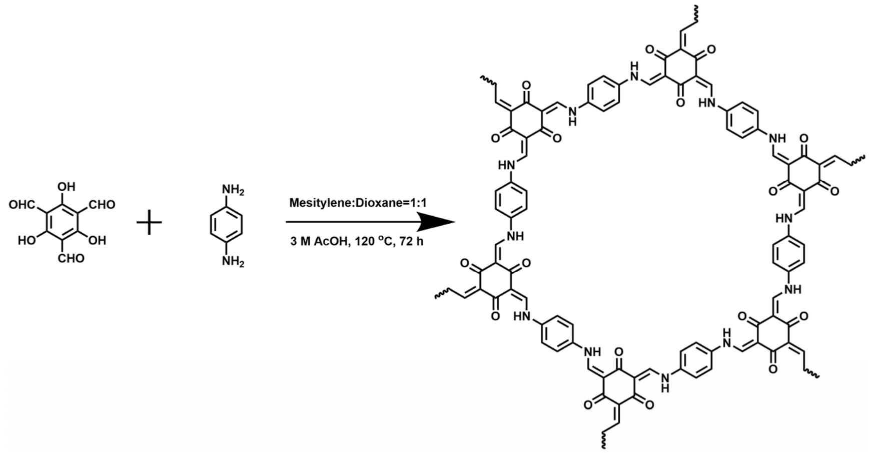

Scheme S1. Synthetic route of TpPa COF

Synthesis of SCOF-1

Tp (63 mg, 0.3 mmol), Pa-SO_3H (85 mg, 0.45 mmol), 1,4-dioxane (20 mL) and aqueous acetic acid (3 M, 2 mL) were mixed under ultrasonication (20 min) to obtain a homogeneous dispersion. The mixture was transferred into an autoclave and heated to 120 °C for 72 h. The obtained red solid was washed successively with ethanol, deionized water and ethanol for three times, and then dried at 50 °C under vacuum for 12 h.[S7]

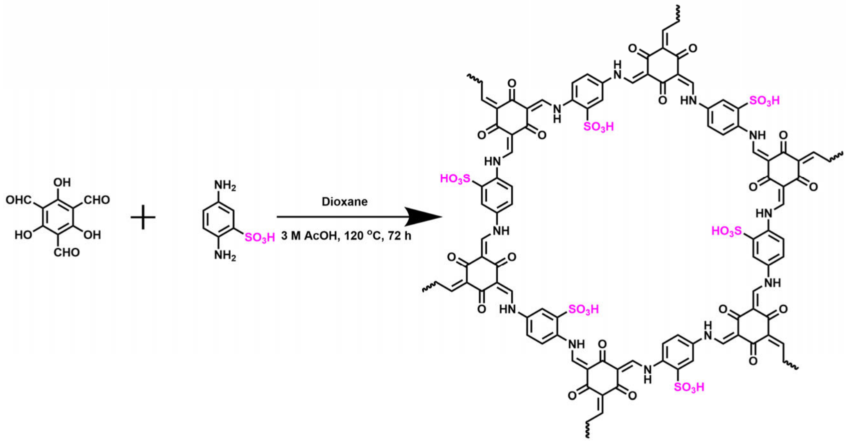

Scheme S2. Synthetic route of SCOF-1

Synthesis of SCOF-3

Tp (42 mg, 0.2 mmol) and BD-$(SO_3H)_2$ (103.3 mg, 0.3 mmol) were added to a Pyrex tube containing a mesitylene/1,4-dioxane (3/1 (v/v)) mixed solvent (4 mL) with 6 M AcOH (0.5 mL), which was then sonicated for 20 min to get a homogeneous mixture. The reaction mixture was flash-frozen under liquid nitrogen and degassed by three freeze–pump–thaw cycles. Then, the tube was sealed and heated at 120 °C for 72 h. The resultant precipitate was collected by filtration and washed with ethanol, acetone, and deionized water for three times. After drying under vacuum at 120 °C for 12 h, the SCOF-3 was obtained as deep red powders.[S8]

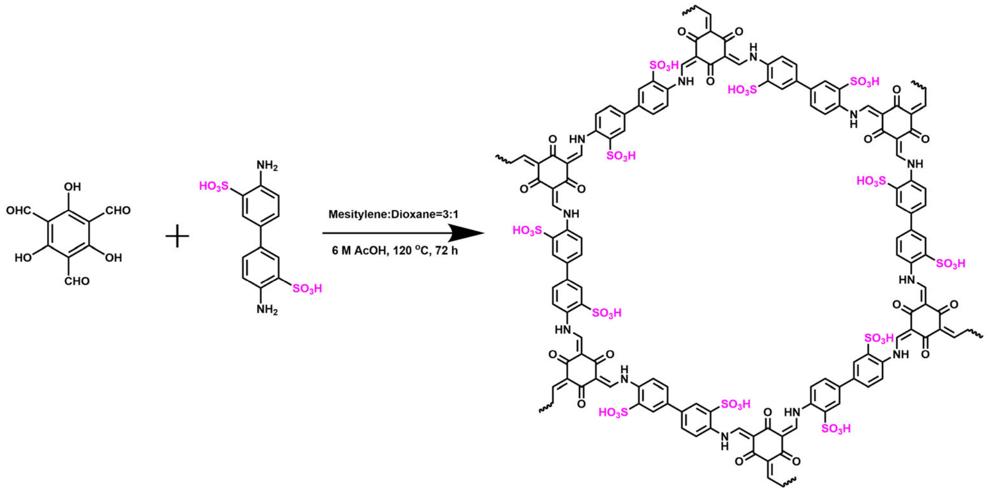

Scheme S3. Synthetic route of SCOF-3

Synthesis of SCOF-4

TFPB (20 mg, 0.05 mmol) and BDSA (30 mg, 0.075 mmol) were mixed together

inside a 15 mL Pyrex tube in the presence of mesitylene/1,4-dioxane (1/1 (v/v)) mixed solvent (4 mL). Then 0.1 mL of AcOH was added. the system was flash-frozen under the environment of liquid nitrogen and degassed via three freeze–pump–thaw cycles. The reactor was sealed off and the resulting mixture was heated at 120 °C for 72 h. Upon the accomplishment of the condensation reaction, the precipitate was collected by centrifugation and extracted by Soxhlet extraction for 48 h with anhydrous THF until the trapped guest molecules and solvents were completely removed. Eventually, the light yellow powder denoted as SCOF-4 was obtained after vacuum-drying overnight at 60 °C for 12 h.[S9]

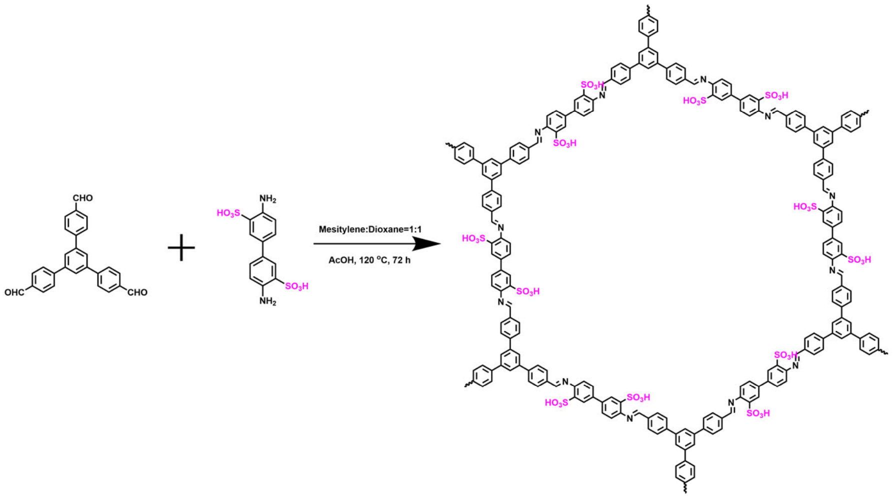

Scheme S4. Synthetic route of SCOF-4

Tryptic Digestion of Proteins

Proteins (HRP, IgG or BSA) were dissolved in NH_4HCO_3 solution (50 mM, pH ~ 10) with a final concentration of 1 μg μL^{-1} for HRP before the solution was heated at 100 °C and 10 μg μL^{-1} for BSA. Then the mixed solution were followed by digestion with trypsin with a concentration ratio of 50:1 (protein versus trypsin) respectively. Thereafter, they were incubated in a 37 °C water bath for 20 h. Finally, the samples were lyophilized and stored at −20 °C.

Human serum that used in the complex samples analysis were obained from patients with ovarian cancer from HUST (Huazhong University of Science and Technology) before pretreatment. Also, we were obtained the approval of patients and the ethical constent from the local committee. Human serum samples were collected from 6 donors

(3 healthy volunteers and 3 patients with ovarian cancer). After centrifugation at 10,000 g for 3 min, the supernatant was diluted 100-fold with the loading buffer and then heated at 95 °C for 10 min, reduced by 20 mM DTT at 56 °C for 45 min, and alkylated by 40 mM IAA at 37 °C in the dark for 45 min. Trypsin was then added into the samples (trypsin: protein = 1:50, w/w) and incubated at 37 °C for 20 h. Finally, the peptides were desalted, lyophilized, and collected for further use. Finally, the samples were lyophilized and stored at −20 °C.

Section 3. Supporting Figures

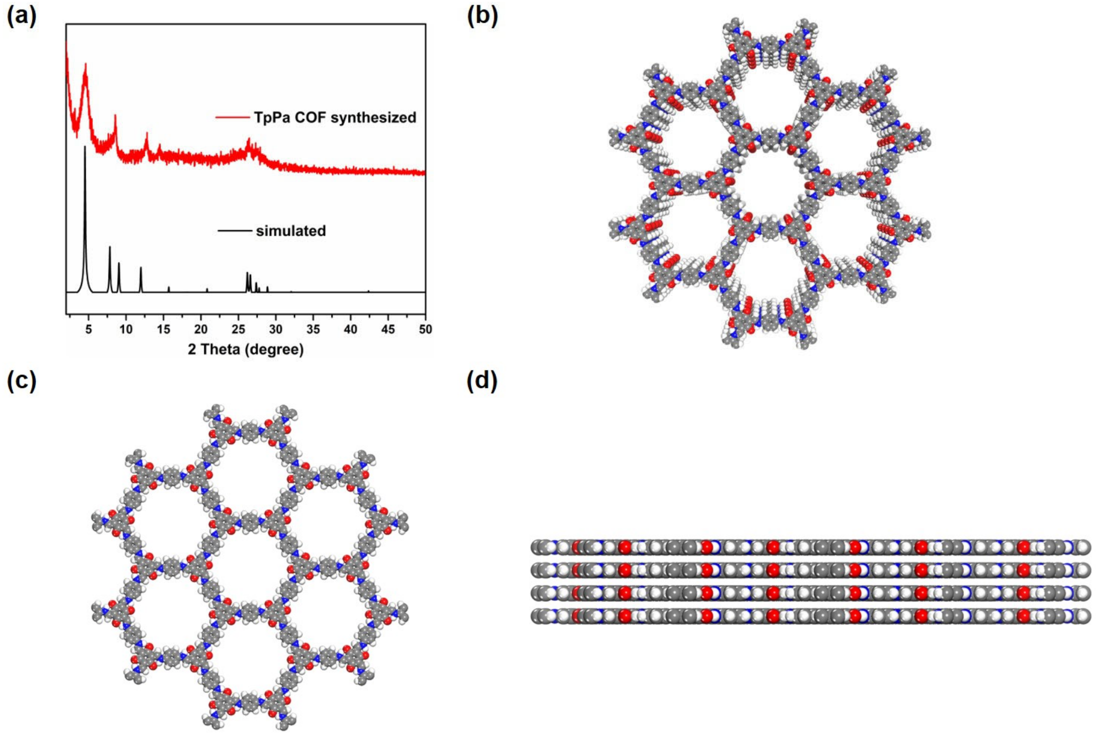

Figure S1. (a) Corresponding PXRD patterns for TpPa COF. (b) The eclipsed stacking mode of TpPa COF. (c) Top view and (d) Side view of the TpPa COF.

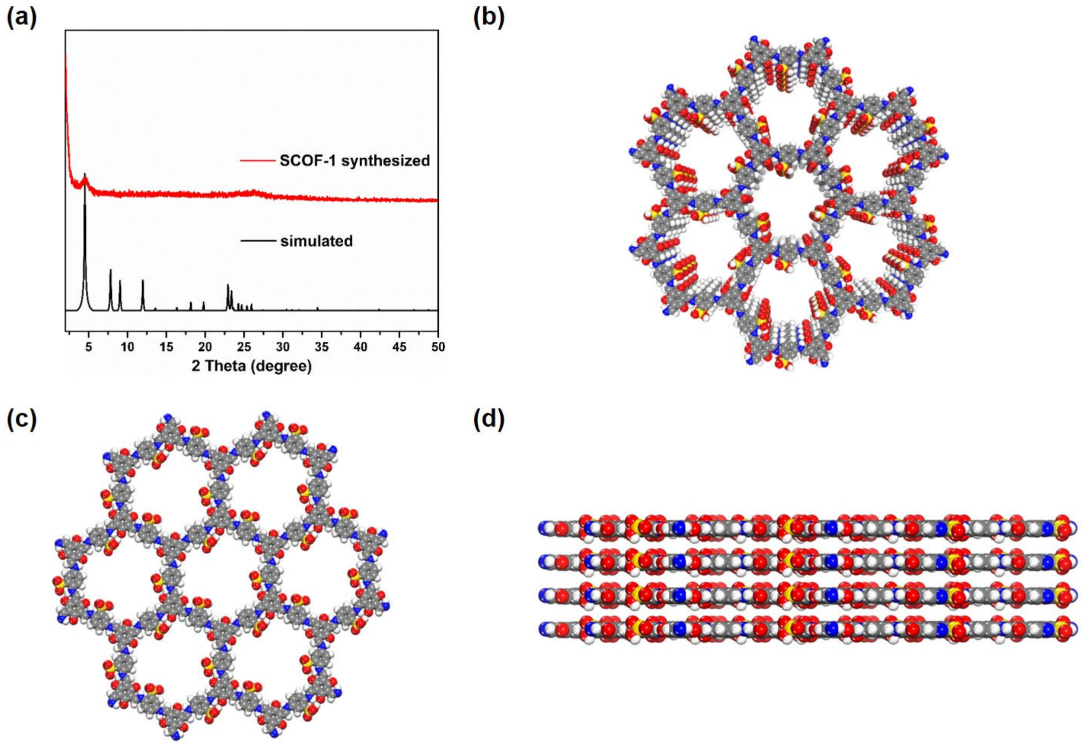

Figure S2. (a) Corresponding PXRD patterns for SCOF-1. (b) The eclipsed stacking mode of SCOF-1. (c) Top view and (d) Side view of the SCOF-1.

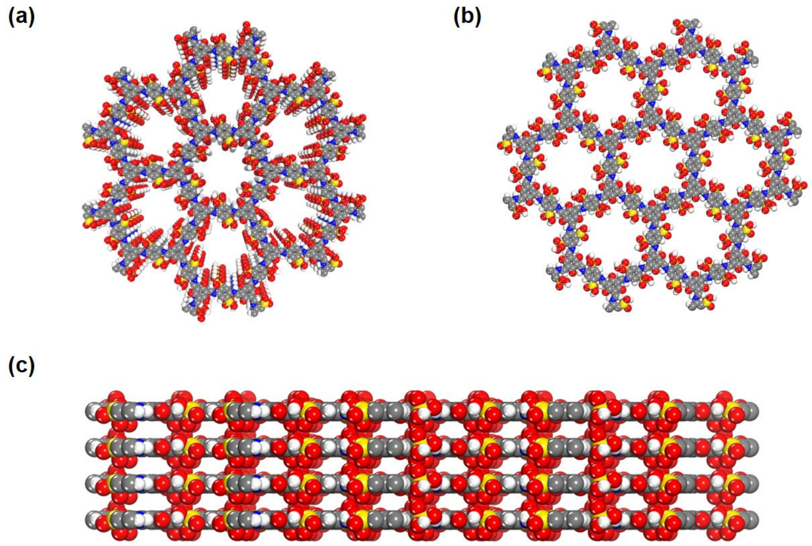

Figure S3. (a) The eclipsed stacking mode of SCOF-2. (b) Top view and (c) Side view of the SCOF-2.

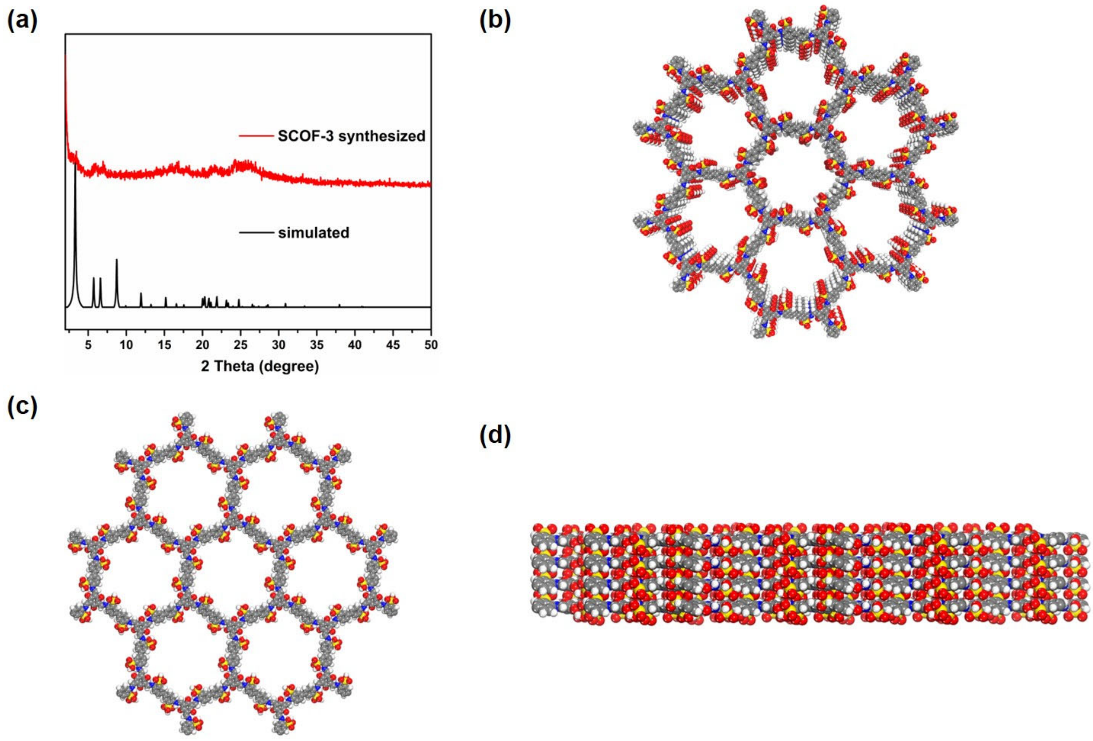

Figure S4. (a) Corresponding PXRD patterns for SCOF-3. (b) The eclipsed stacking mode of SCOF-3. (c) Top view and (d) Side view of the SCOF-3.

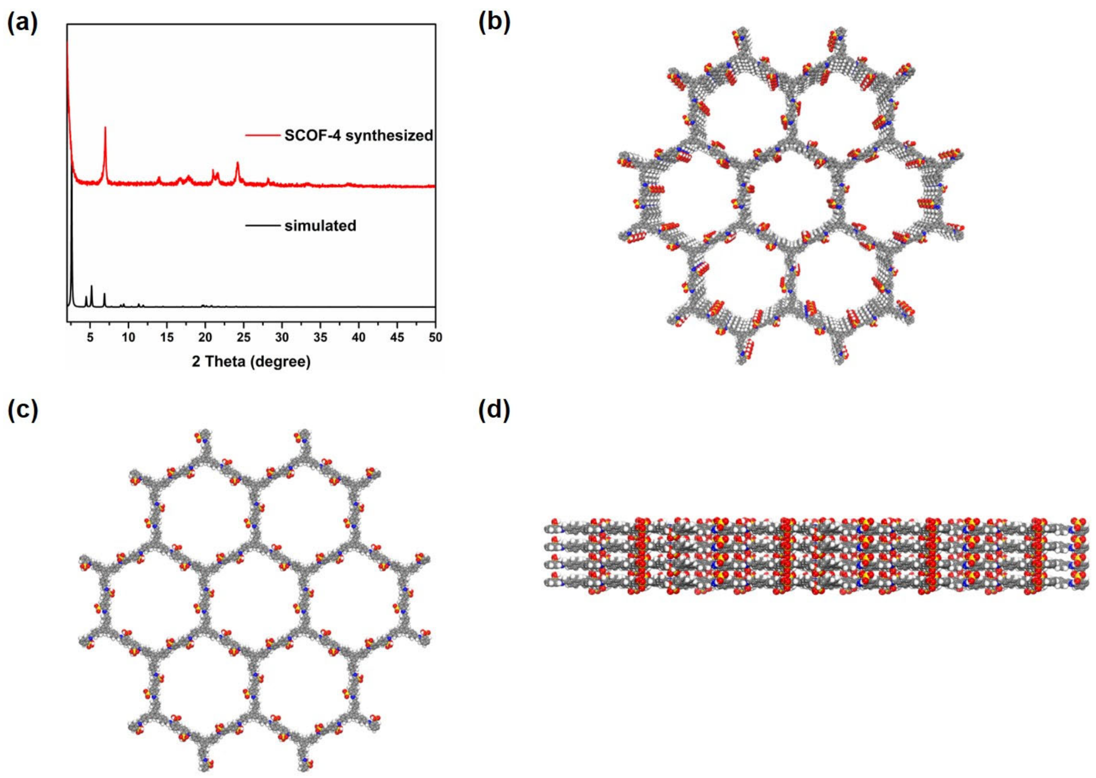

Figure S5. (a) Corresponding PXRD patterns for SCOF-4. (b) The eclipsed stacking mode of SCOF-4. (c) Top view and (d) Side view of the SCOF-4.

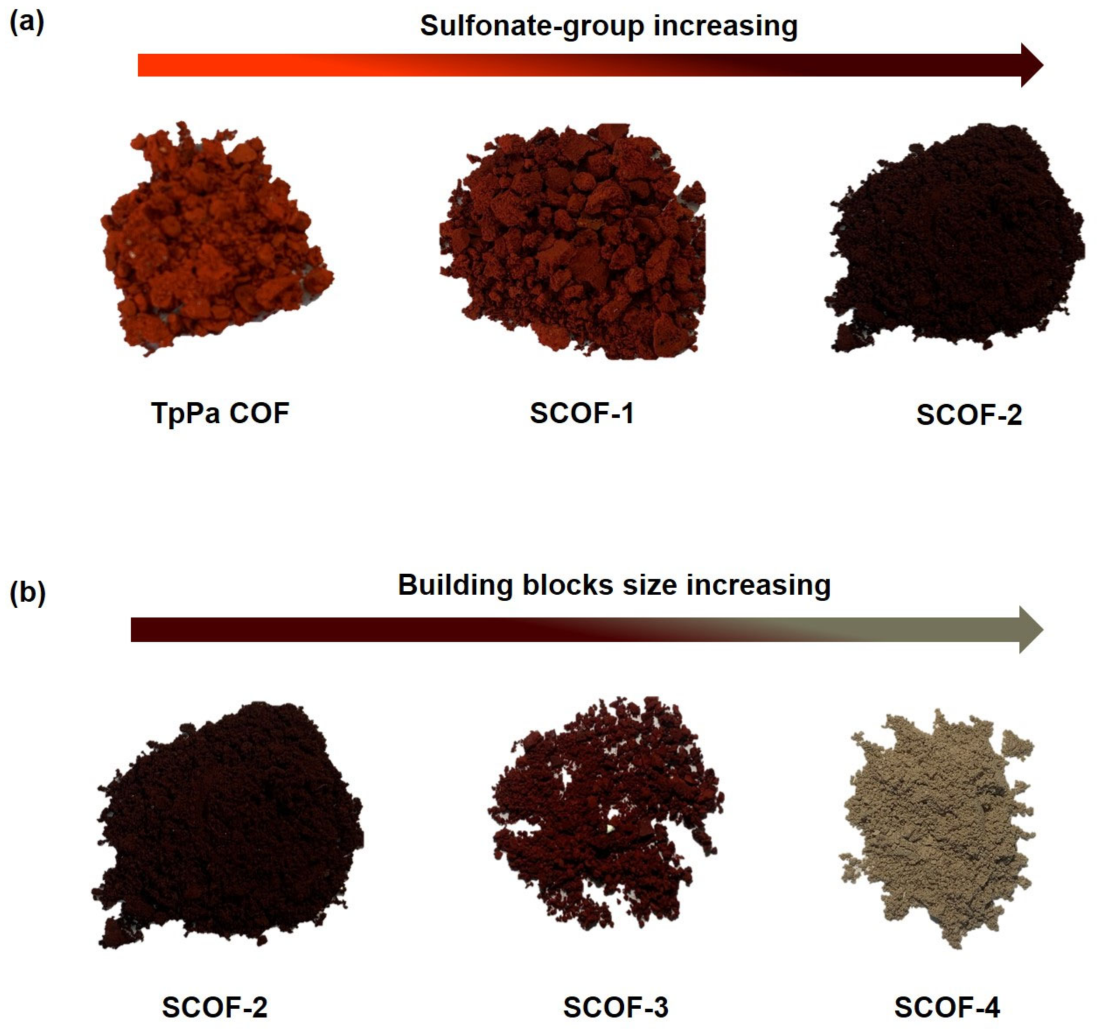

Figure S6. Optical photos of the synthesized (a) TpPa COF, SCOF-1 and SCOF-2; (b) SCOF-2, SCOF-3 and SCOF-4.

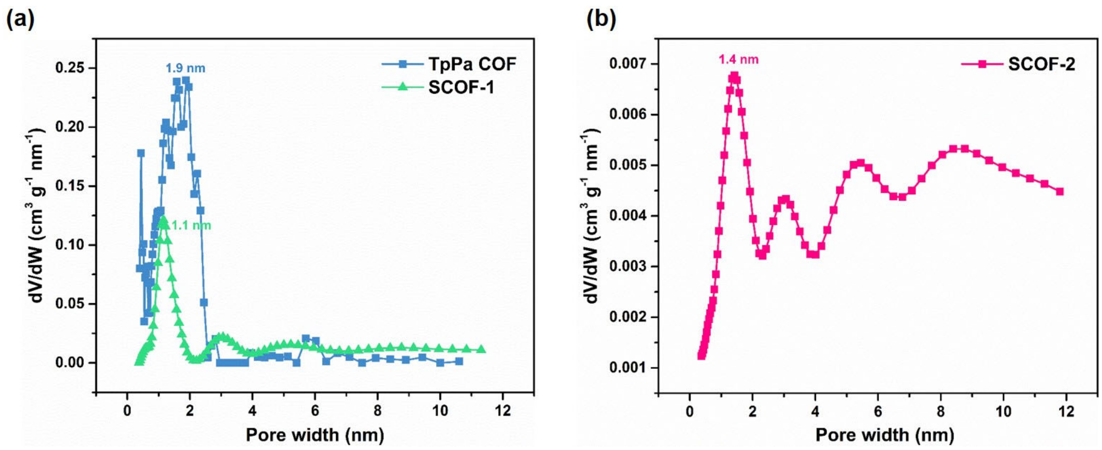

Figure S7. Pore size distribution (PSD) of (a) TpPa COF and SCOF-1; (b) SCOF-2.

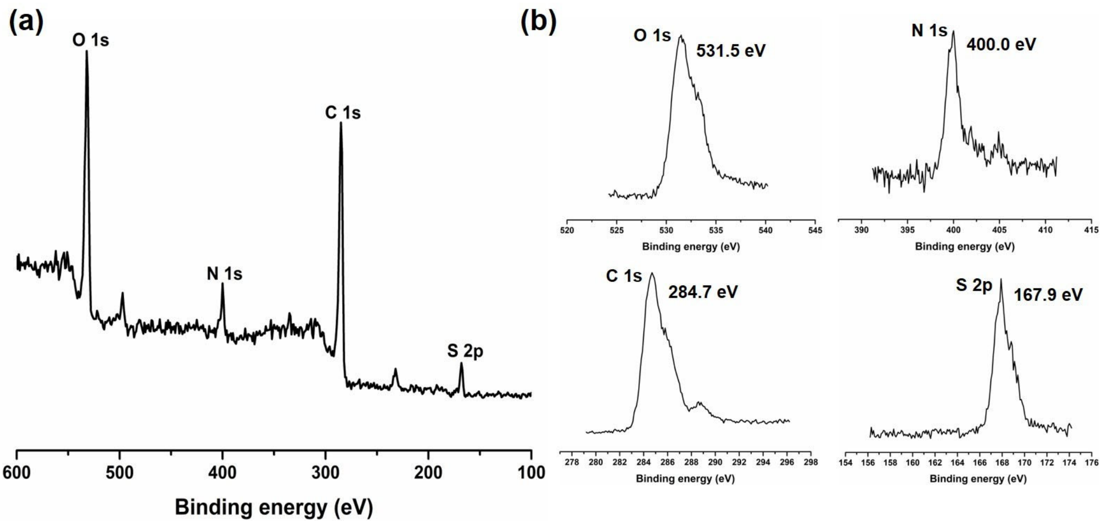

Figure S8. XPS spectrum of SCOF-2.

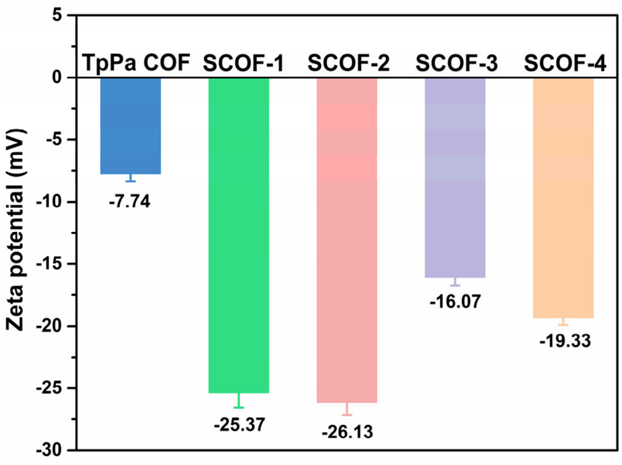

Figure S9. Zeta potential value of TpPa COF, SCOF-1, SCOF-2, SCOF-3 and SCOF-4.

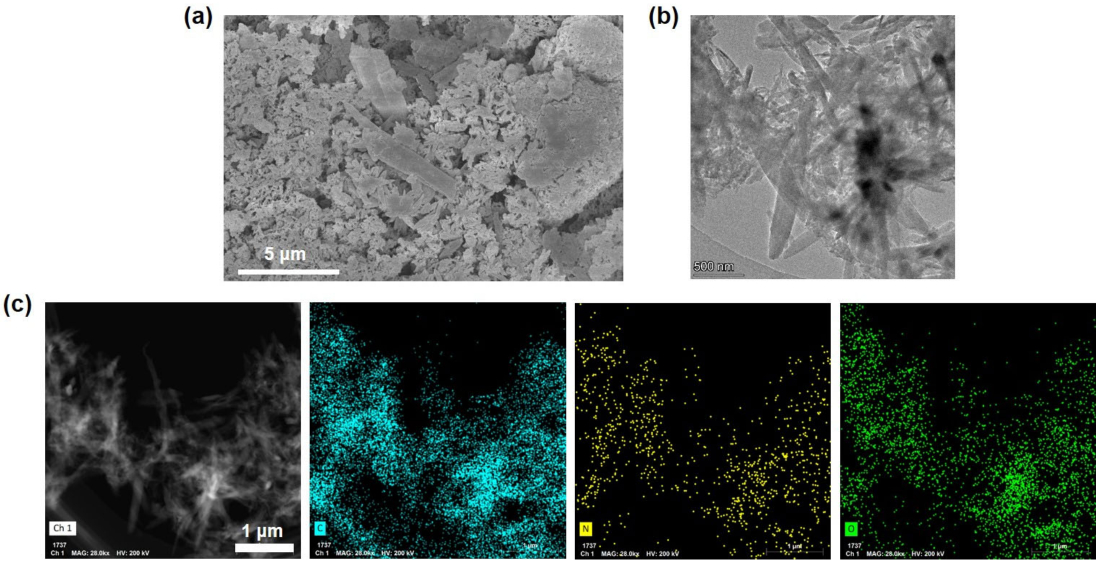

Figure S10. (a) SEM image, (b) TEM image, and (c) HAADF-STEM image with the C, N and O elemental mapping images of TpPa COF.

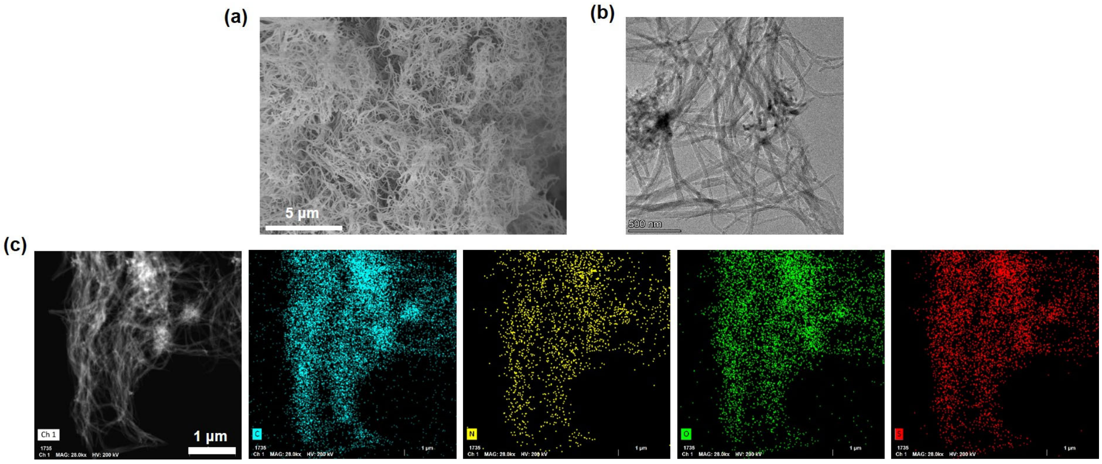

Figure S11. (a) SEM image, (b) TEM image, and (c) HAADF-STEM image with the C, N, O and S elemental mapping images of SCOF-1.

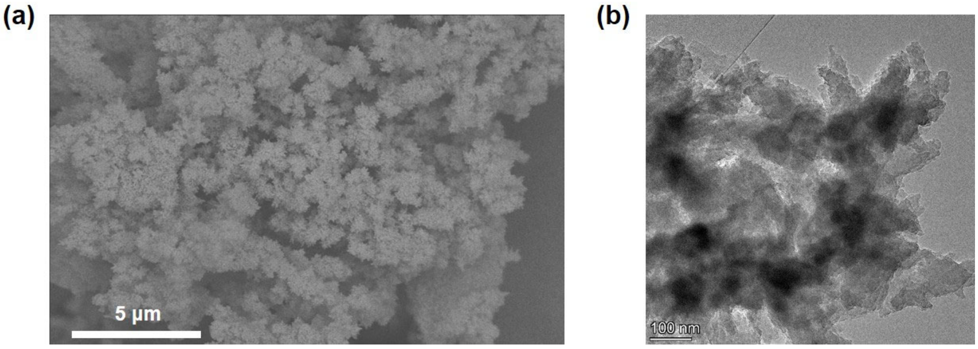

Figure S12. (a) SEM image and (b) TEM image of SCOF-2.

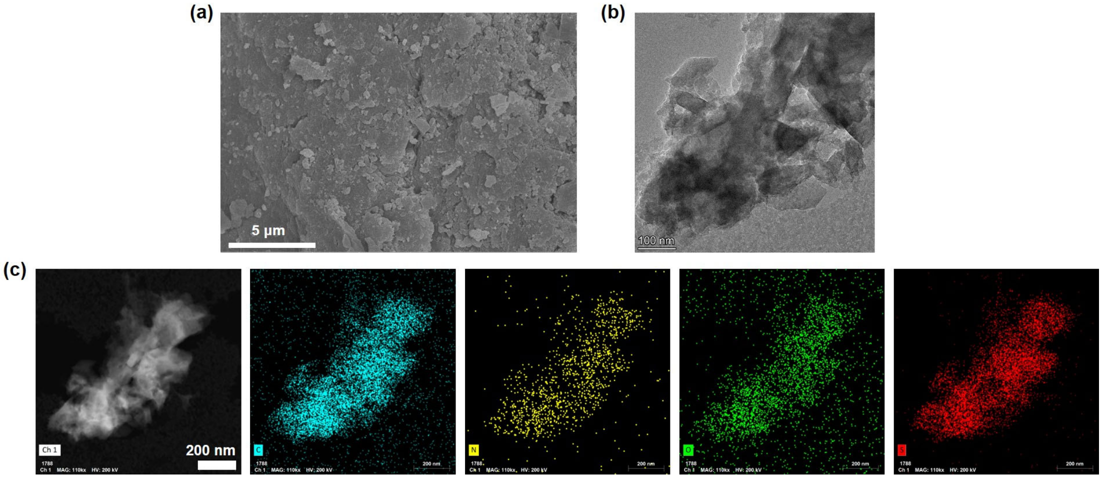

Figure S13. (a) SEM image, (b) TEM image, and (c) HAADF-STEM image with the C, N, O and S elemental mapping images of SCOF-3.

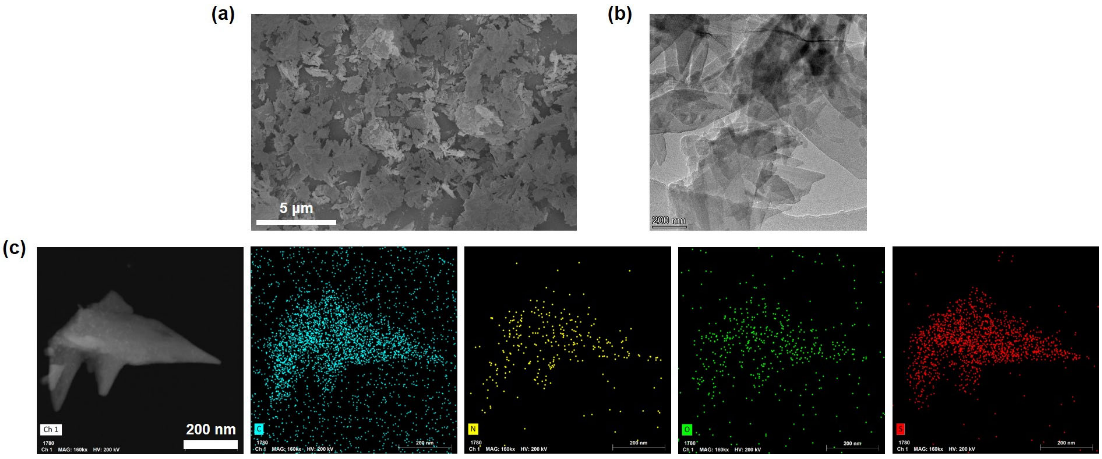

Figure S14. (a) SEM image, (b) TEM image, and (c) HAADF-STEM image with the C, N, O and S elemental mapping images of SCOF-4.

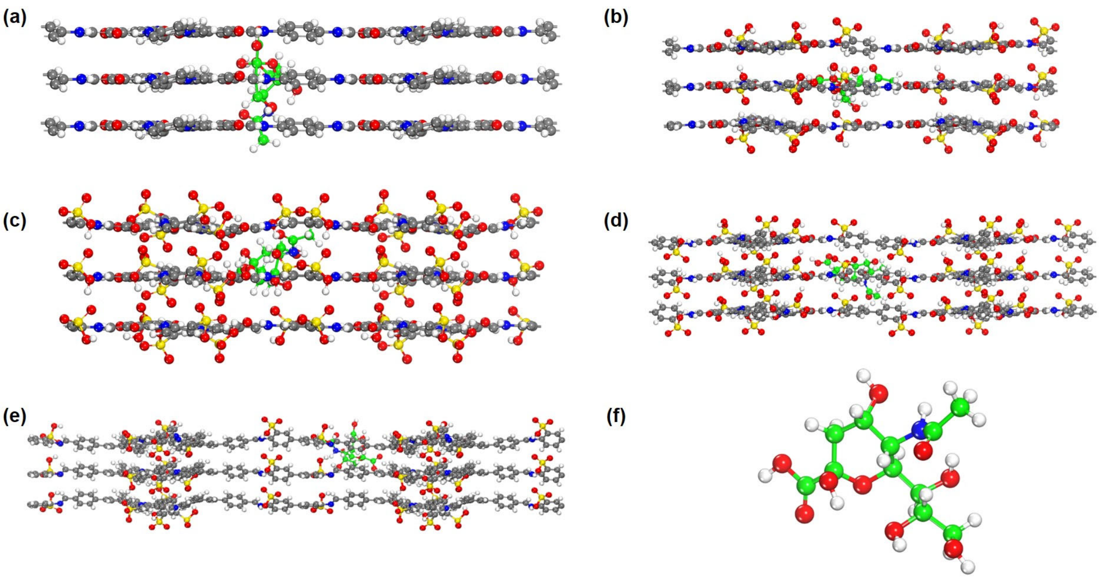

Figure S15. Schmetic illustration of the side view possible adsorption modes between (a) TpPa COF, (b) SCOF-1, (c) SCOF-2, (d) SCOF-3, and (e) SCOF-4 and Neu5Ac, respectively. (f) The sialylated glycan structure of the Neu5Ac.

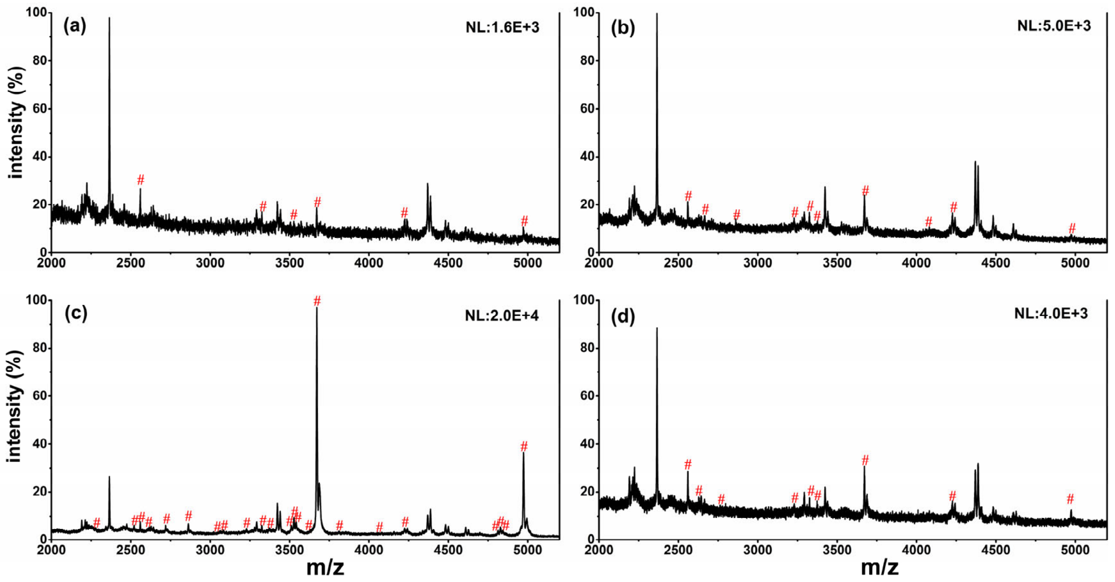

Figure S16. MALDI-TOF MS of glycopeptides from HRP tryptic digest enriched by SCOF-2 with different concentrations of ACN in loading buffer: (a) 80% ACN, (b) 85% ACN, (c) 90% ACN, and (d) 95% ACN. Glycopeptide peaks were marked with "#". NL: Normalized Level.

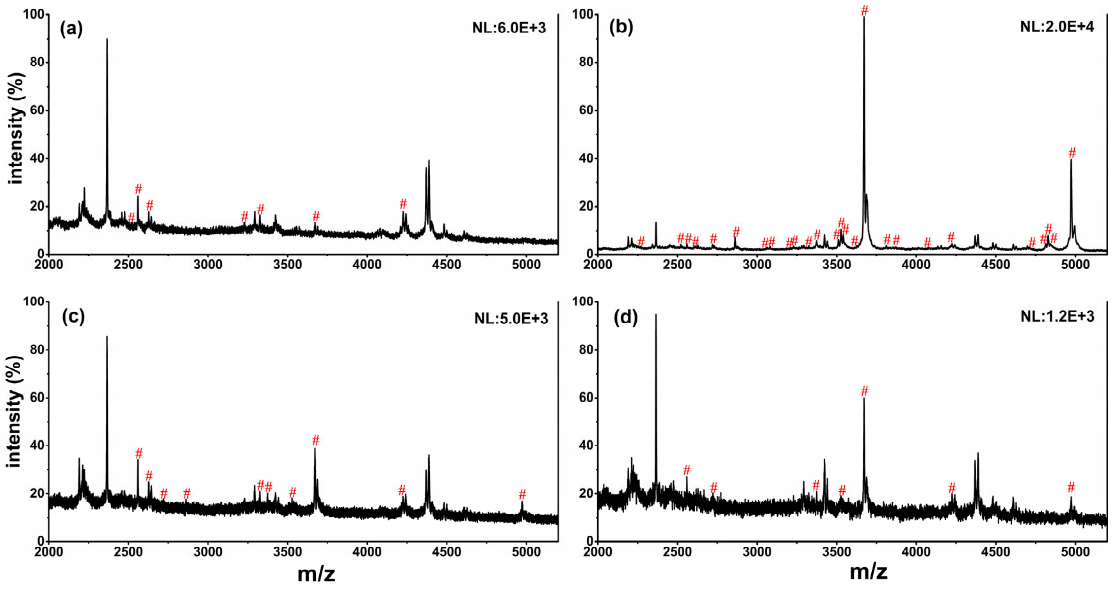

Figure S17. MALDI-TOF MS of glycopeptides from HRP tryptic digest enriched by SCOF-2 with different concentrations of TFA in loading buffer: (a) 0.05% TFA, (b) 0.1% TFA, (c) 0.5% TFA, and (d) 1.0% TFA. Glycopeptide peaks were marked with "#". NL: Normalized Level.

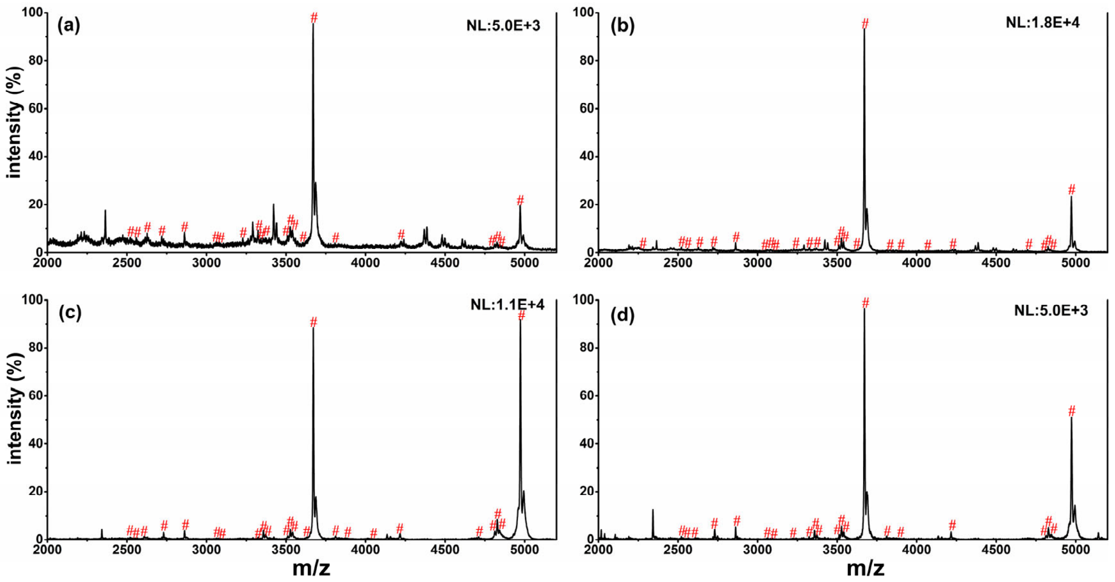

Figure S18. MALDI-TOF MS of glycopeptides from HRP tryptic digest enriched by SCOF-2 with different concentrations of TFA in elution buffer: (a) 0.05% TFA, (b) 0.1% TFA, (c) 0.5% TFA, and (d) 1.0% TFA. Glycopeptide peaks were marked with "#". NL: Normalized Level.

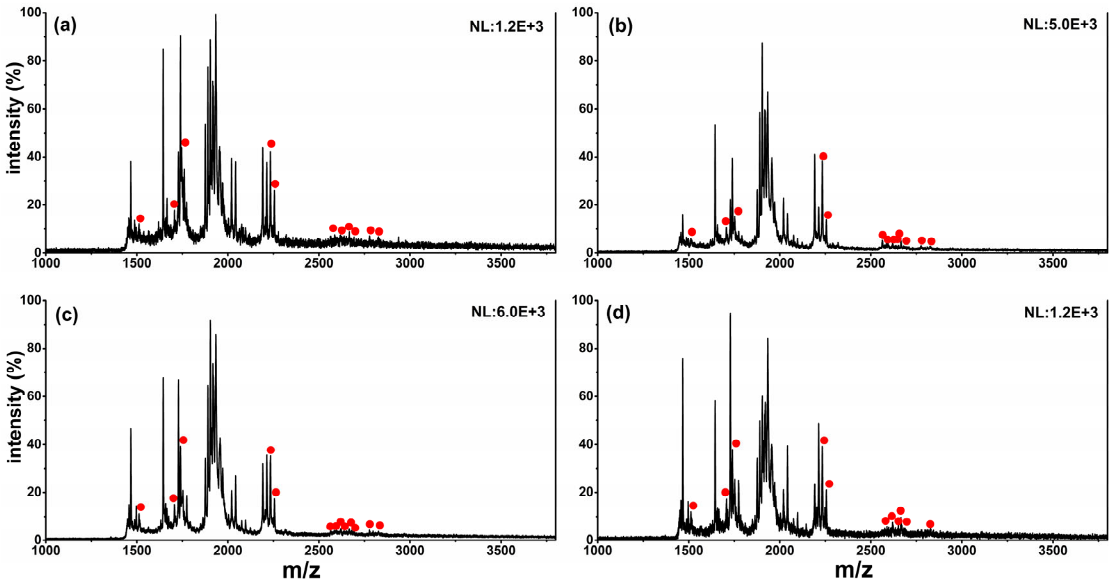

Figure S19. MALDI-TOF MS of glycopeptides from IgG tryptic digest enriched by SCOF-2 with different concentrations of ACN in loading buffer: (a) 80% ACN, (b) 85% ACN, (c) 90% ACN, and (d) 95% ACN. Glycopeptide peaks were marked with "#". NL: Normalized Level.

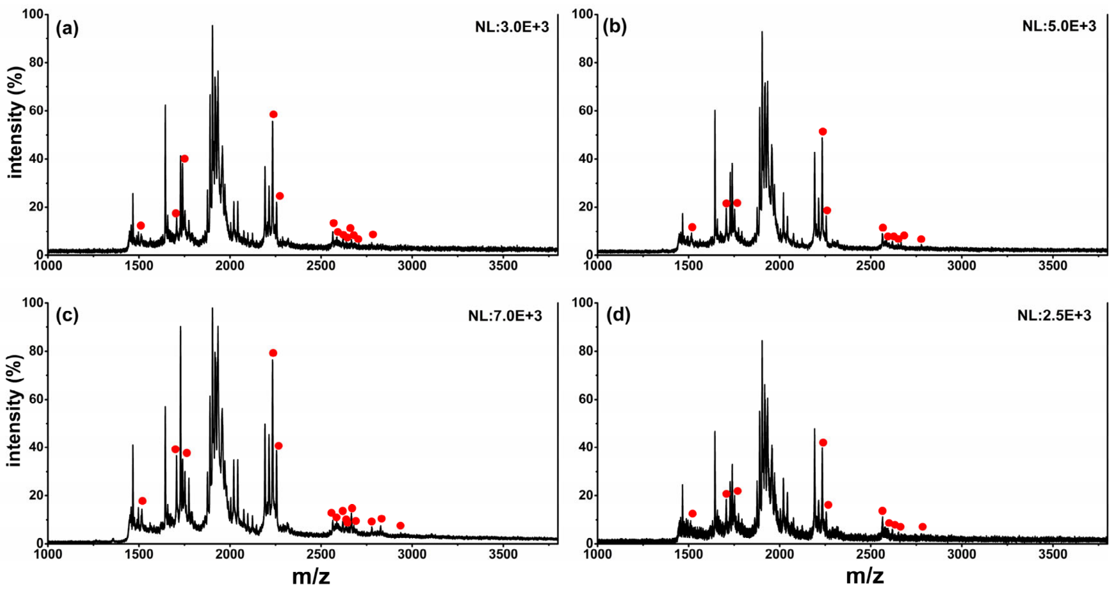

Figure S20. MALDI-TOF MS of glycopeptides from IgG tryptic digest enriched by SCOF-2 with different concentrations of TFA in loading buffer: (a) 0.5% TFA, (b) 1.0% TFA, (c) 1.5% TFA, and (d) 2.0% TFA. Glycopeptide peaks were marked with "#". NL: Normalized Level.

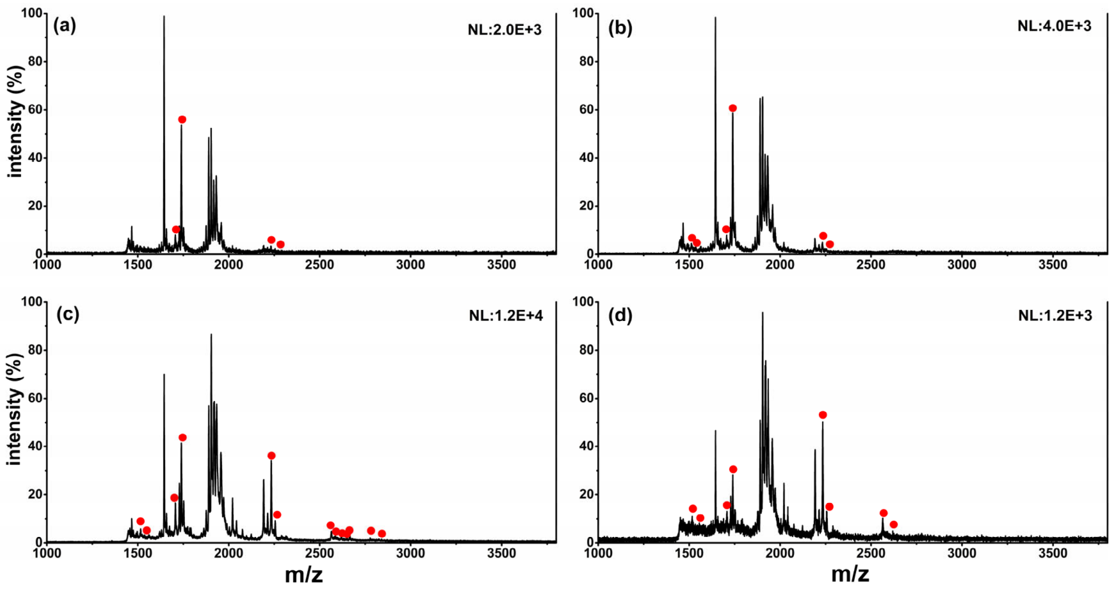

Figure S21. MALDI-TOF MS of glycopeptides from IgG tryptic digest enriched by SCOF-2 with different concentrations of TFA in elution buffer: (a) 0.05% TFA, (b) 0.1% TFA, (c) 0.5% TFA, and (d) 1.0% TFA. Glycopeptide peaks were marked with "#". NL: Normalized Level.

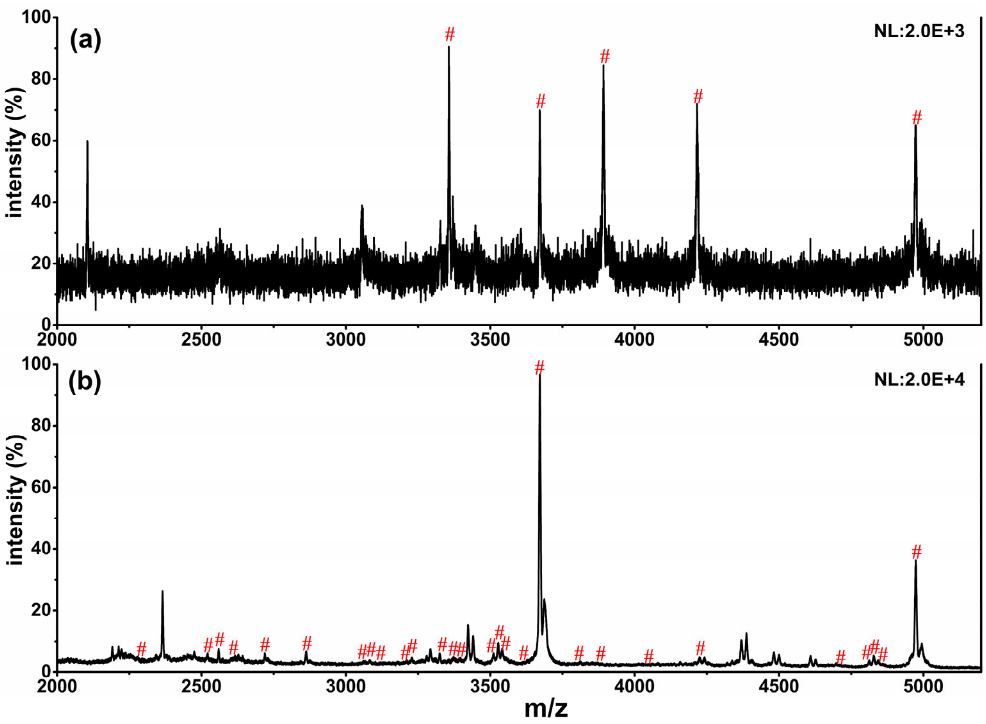

Figure S22. MALDI-TOF MS of glycopeptides from HRP tryptic digest: (a) direct analysis, (b) after enriched by SCOF-2. Glycopeptide peaks were marked with "#". NL: Normalized Level.

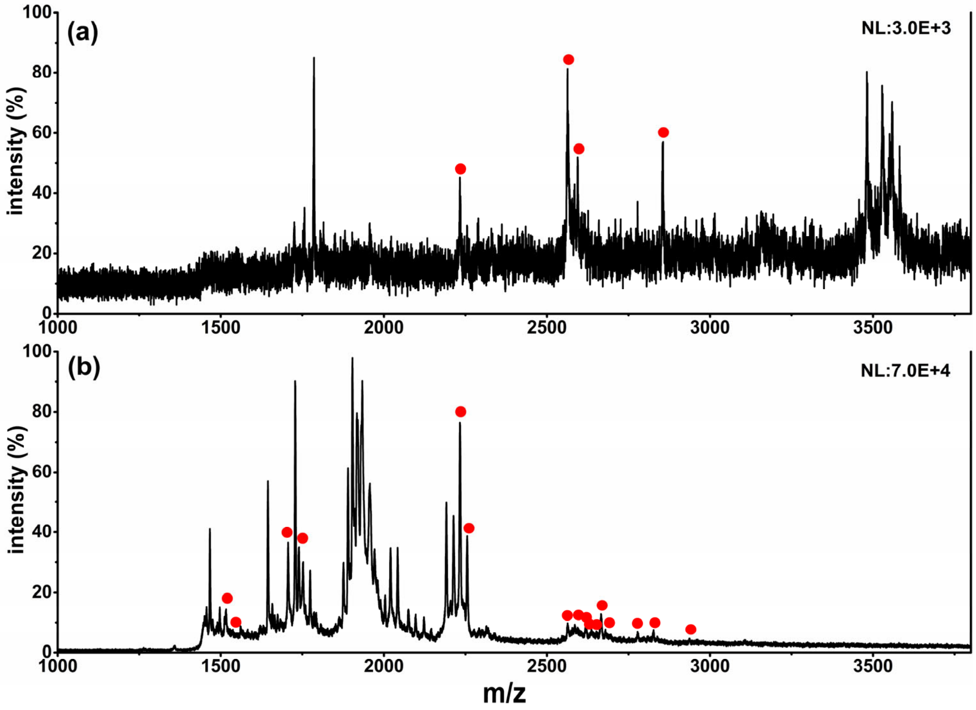

Figure S23. MALDI-TOF MS of glycopeptides from IgG tryptic digest: (a) direct analysis, (b) after enriched by SCOF-2. Glycopeptide peaks were marked with "#". NL: Normalized Level.

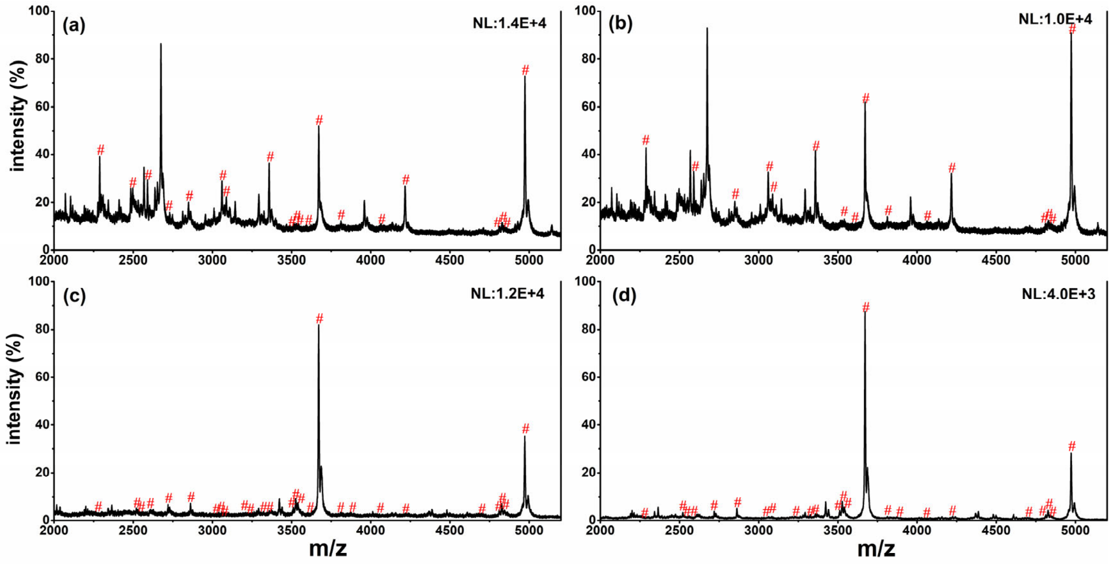

Figure S24. MALDI-TOF MS of glycopeptides from HRP and BSA tryptic digests enriched by SCOF-2 with different molar ratios of (a) 1:10 and (b) 1:100. MALDI-TOF MS of glycopeptides from HRP tryptic digest enriched by SCOF-2 with different concentrations of (c) 10 fmol μL^{-1} and (d) 1.0 fmol μL^{-1}. Glycopeptide peaks were marked with "#". NL: Normalized Level.

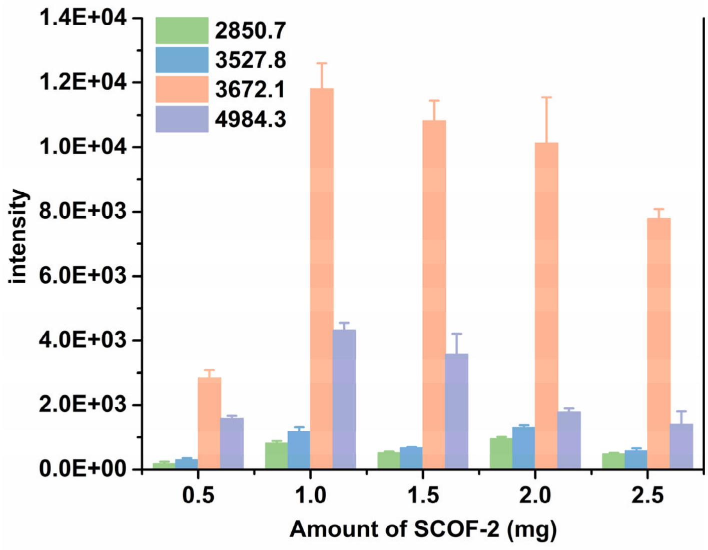

Figure S25. Peak intensity of four most intense glycopeptides from the HRP tryptic digest by different amounts of SCOF-2.

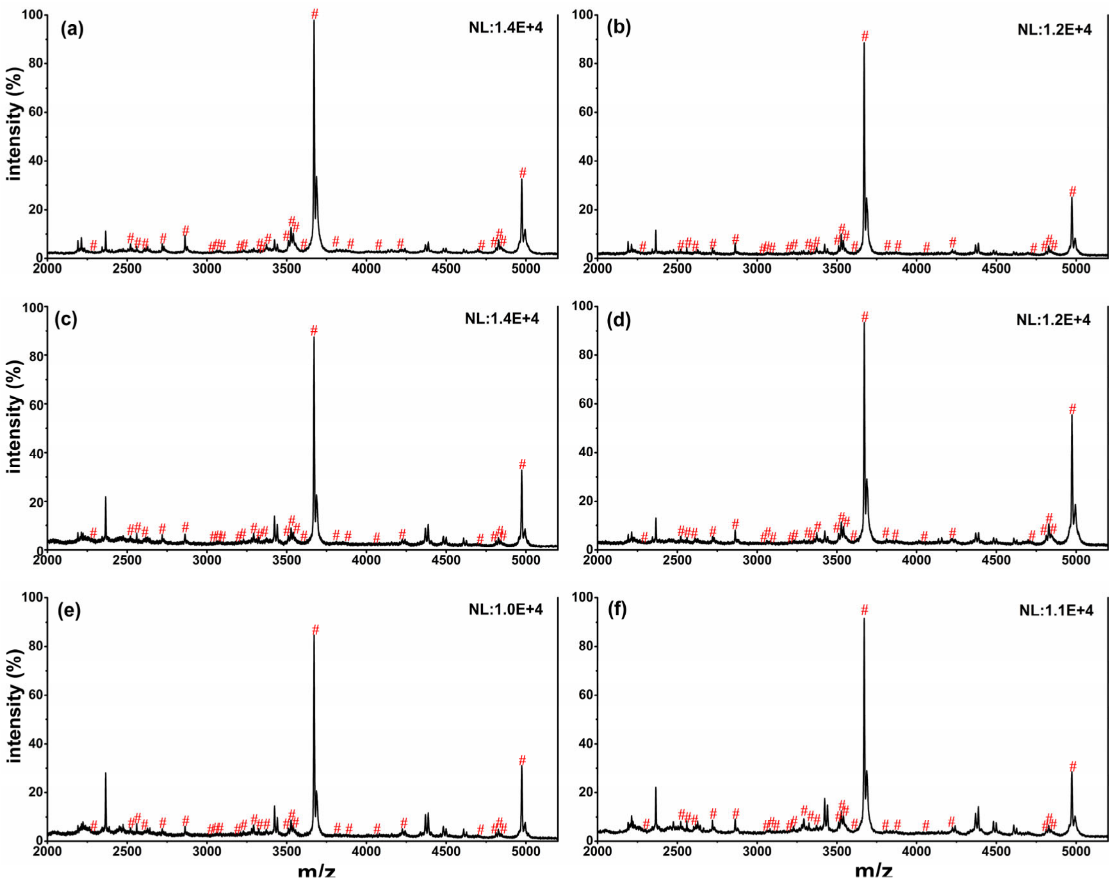

Figure S26. The cycling performance of the SCOF-2: (a) the first time, (b) the second time, (c) the third time, (d) the fourth time, (e) the fifth time and (f) after enrichment with the SCOF-2 placed for two weeks. Glycopeptide peaks were marked with "#". NL: Normalized Level.

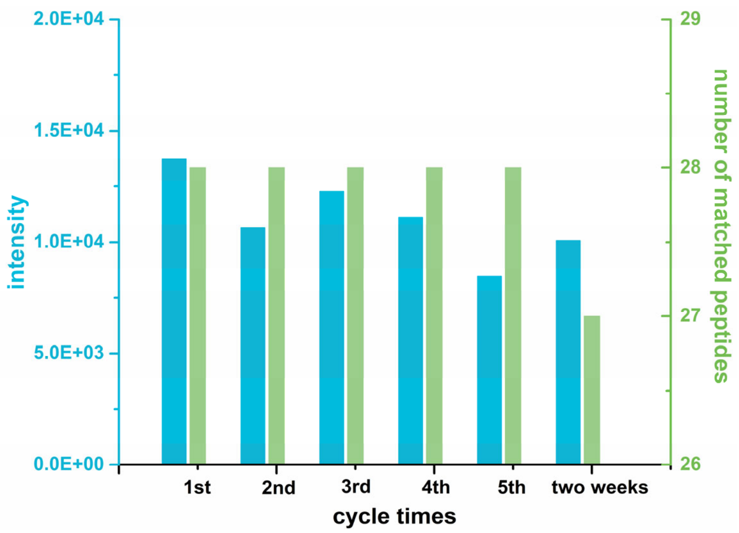

Figure S27. Cycling performance of the SCOF-2 for glycopeptides enrichment.

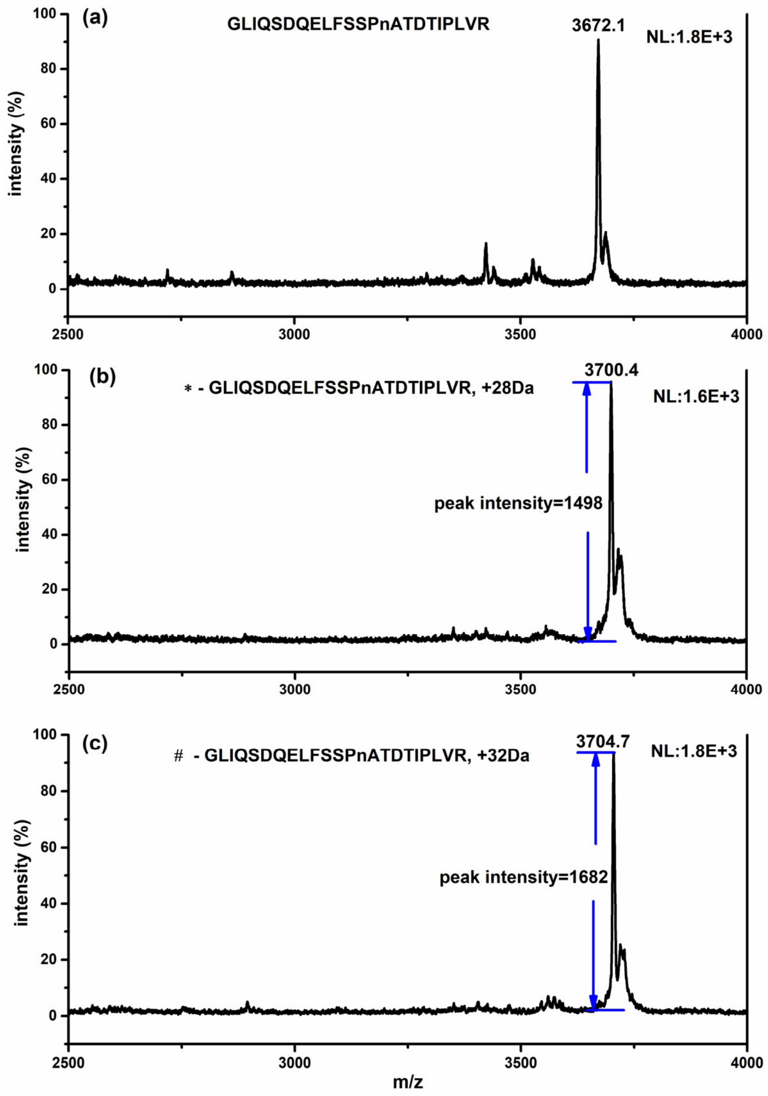

Figure S28. The experiment of the recovery of the light- and heavy dimethyl labeling deglycosylated peptides from HRP tryptic digest and the labeling reactions were as follows, recovery was calculated from the measurements of 1:1 mixture of the H-labeled and D-labeled of the m/z = 3672.1 peaks.

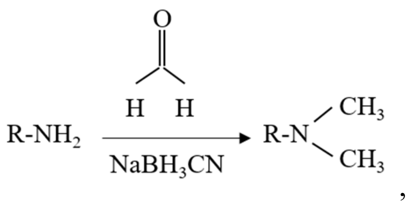

+28Da, light-labeled

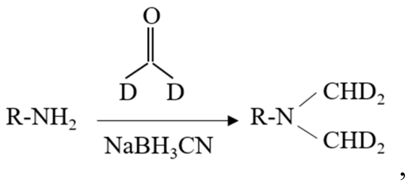

+32Da, heavy-labeled

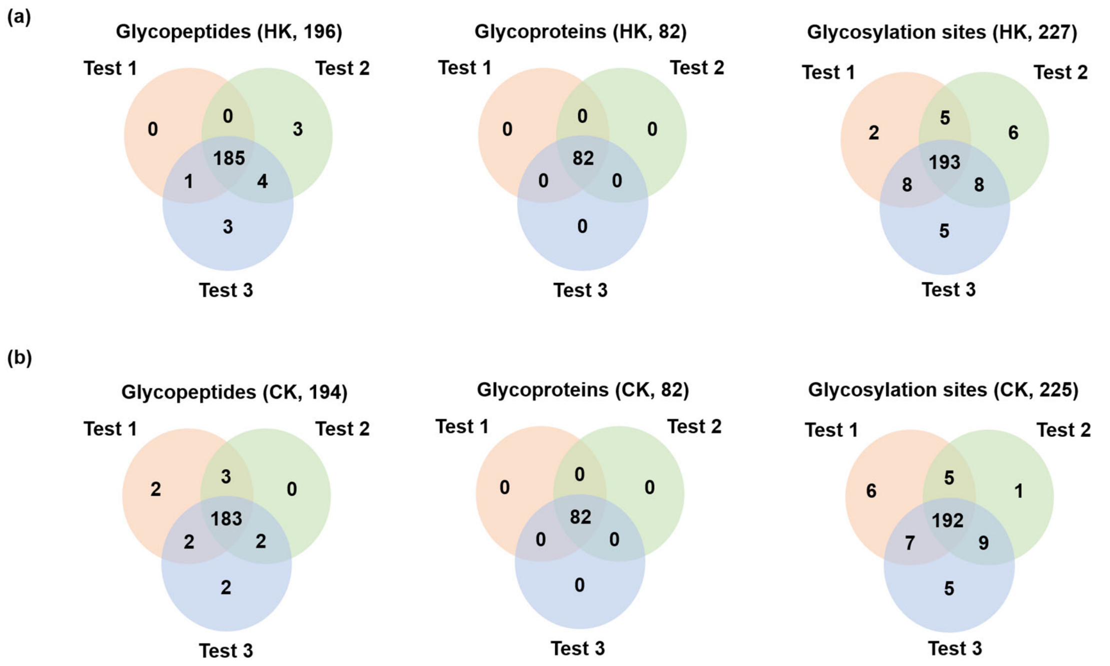

Figure S29. Venn diagram of glycopeptides, glycoproteins and glycosylation sites identified in each isolated serum samples of (a) ovarian cancer patients, group HK and (b) healthy controls, group CK.

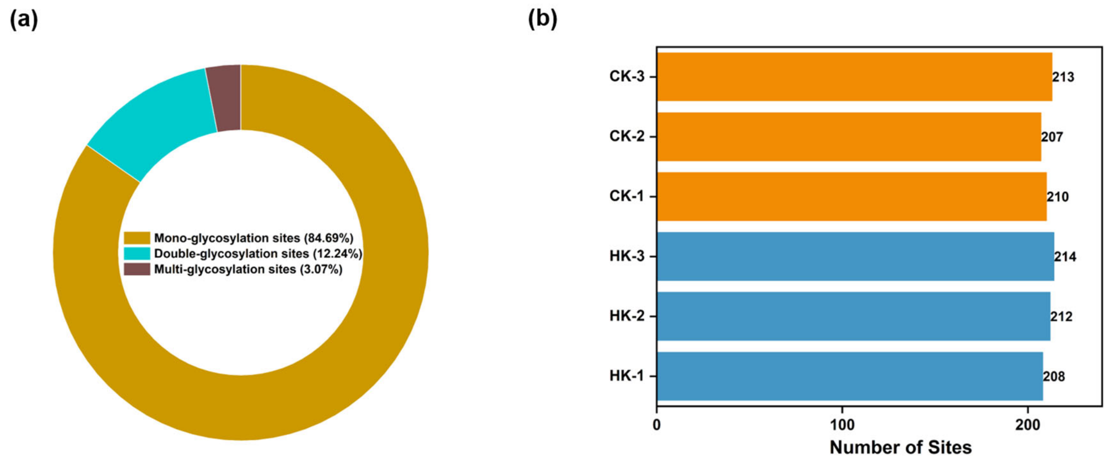

Figure S30. (a) Distribution of mono-, double-, and multi- glycosylation sites and (b) detailed information of identified glycosylation sites observed from ovarian cancer patients, group HK and healthy controls, group CK.

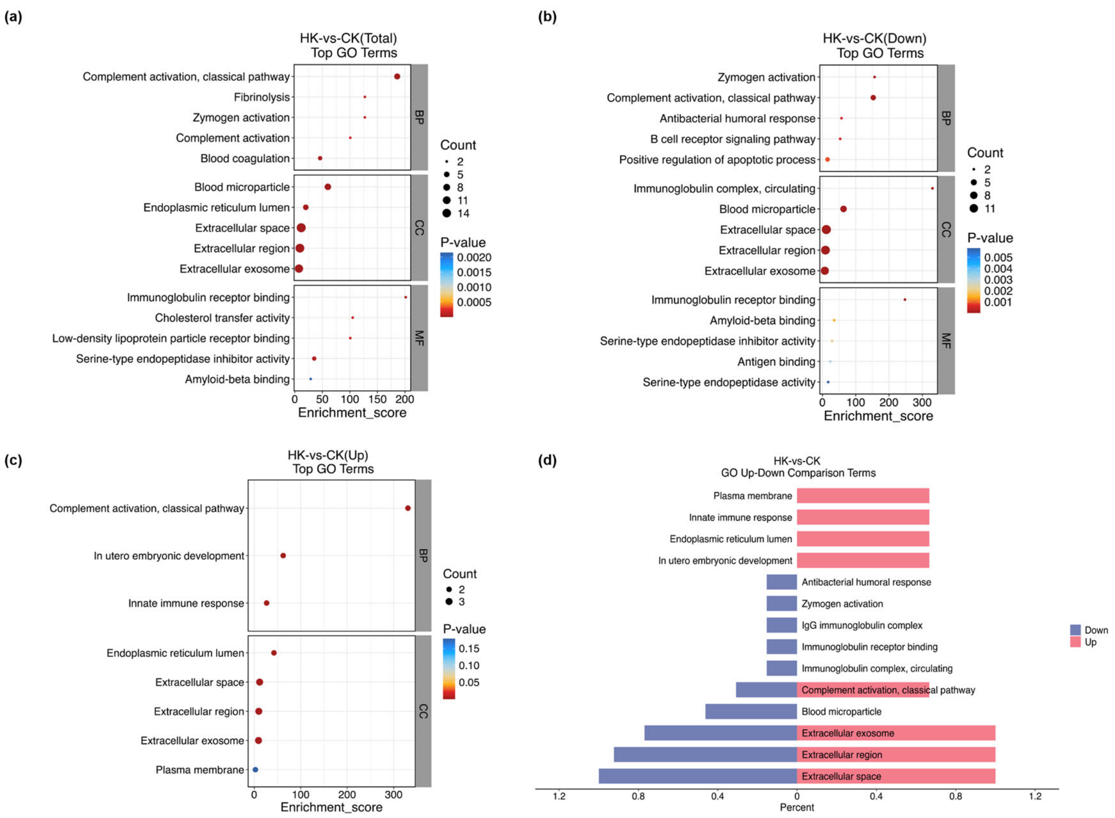

Figure S31. GO enrichment of glycoproteins of human serum (a) total, (b) down-regulated, (c) up-regulated and (d) up-down comparison from ovarian cancer patients, group HK and healthy controls, group CK.

Section 4. Supporting Tables

Table S1. The detailed information of glycopeptides identified from HRP tryptic digest by SCOF-2. N# denotes the N-linked glycosylation site.

NO.	Glycan composition	Peptide sequence	Observed m/z
H1	XylMan2FucGlcNAc2	SILLDN#TTSFR	2286.5
H2	FucGlcNAc	SFAnSTQTFFNAFVEAMDR	2531.9
H3	XylMan3FucGlcNAc2	SSPnATDTIPLVR	2541.7
H4	XylMan3GlcNAc2	MGnITPLTGTQGQIR	2611.6
H5	FucGlcNAc	GLIQSDQELFSSPnATDTIPLVR	2704.5
H6	XylMan2GlcNAc2	SFAnSTQTFFNAFVEAMDR	2850.7
H7	FucGlcNAc	LHFHDCFVNGCDASILLDnTTSFR	3048.6
H8	XylMan3FucGlcNAc2	GLCPLNGnLSALVDFDLR	3074.7
H9	XylMan3GlcNAc2	SFAnSTQTFFNAFVEAMDR	3089.3
H10	Man3FucGlcNAc2	SFAnSTQTFFNAFVEAMDR	3207.9
H11	XylMan3FucGlcNAc2	QLTPTFYDNSCPnVSNIVR	3221.8
H12	XylMan3FucGlcNAc2	SFAnSTQTFFNAFVEAMDR	3322.3
H13	XylMan3FucGlcNAc2	SFAnSTQTFFNAFVEAMDR	3354.8
H14	Hex2HexNAc2Fuc1	GLIQSDQELFSSPN#ATDTIPLVR	3370.9
H15	Hex2HexNAc2Fuc1Xyl1	GLIQSDQELFSSPN#ATDTIPLVR	3510.1
H16	XylMan3GlcNAc2	GLIQSDQELFSSPnATDTIPLVR	3527.8
H17	Man3FucGlcNAc2	GLIQSDQELFSSPnATDTIPLVR	3541.7
H18	XylMan3FucGlcNAc2	NQCRGLCPLNGnLSALVDFDLR	3607.8
H19	XylMan3FucGlcNAc2	GLIQSDQELFSSPnATDTIPLVR	3672.1
H20	Man2GlcNAc2	LHFHDCFVNGCDASILLDnTTSFRTEK	3812.3
H21	XylMan3FucGlcNAc2	LHFHDCFVNGCDASILLDnTTSFR	3894.6
H22	Hex4HexNAc2Fuc1Xyl1	QLTPTFYDNSCAAVESACPRPN#VSNIVR	4057.6
H23	XylMan3FucGlcNAc2	QLTPTFYDNSCAAVESACPRPnVSNIVR	4221.4
H24	Man3FucGlcNAc2,	LYN#FSNTGLPDPTLN#TTYLQTLR	4720.2

H25	XylMan2FucGlcNAc2,<br>XylMan2GlcNAc2	LYnFSNTGLPDPTLnTTYLQTLR	4821.6
H26	XylMan3FucGlcNAc2,<br>XylMan3GlcNAc2	LYnFSNTGLPDPTLnTTYLQTLR	4838.5
H27	Man3FucGlcNAc2,<br>XylMan3FucGlcNAc2	LYnFSNTGLPDPTLnTTYLQTLR	4852.6
H28	XylMan3FucGlcNAc2,<br>XylMan3FucGlcNAc2	LYnFSNTGLPDPTLnTTYLQTLR	4984.3

Table S2. The detailed information of glycopeptides identified from IgG tryptic digest by SCOF-2. N# denotes the N-linked glycosylation site.

NO.	Glycan composition	Peptide sequence	Observed m/z
I1	HexNAc1Fuc1	EEQFN#STFR	1506.2
I2	HexNAc1Fuc1	EEQYN#STYR	1537.6
I3	HexNAc2Fuc1	EEQFN#STFR	1708.5
I4	HexNAc2Fuc1	EEQYN#STYR	1740.5
I5	Hex2NAc3Fuc1	EEQFN#STFR	2236.3
I6	Hex2HexNAc3Fuc1	EEQYN#STYR	2269.6
I7	Hex4HexNAc3Fuc1	EEQFN#STFR	2560.9
I8	Hex4HexNAc3Fuc1	EEQYN#STYR	2592.9
I9	Hex4HexNAc4	EEQFN#STFR	2617.9
I10	Hex3HexNAc4Fuc1	EEQYN#STYR	2633.9
I11	Hex4HexNAc4	EEQYN#STYR	2650.6
I12	Hex3HexNAc5	EEQFN#STFR	2658.9
I13	Hex3HexNAc5	EEQYN#STYR	2690.9
I14	Hex5HexNAc4	EEQFN#STFR	2780.8
I15	Hex4HexNAc5	EEQFN#STFR	2820.9
I16	Hex5HexNAc4Fuc1	EEQFN#STFR	2926.9

Table S3. Comparison of the enrichment efficiency of the SCOF-2 towards glycopeptides with different hydrophilic materials reported before.

Materials	Number of detected glycopeptides	Limit of detection (fmol μL^{-1})	Enrichment selectivity (HRP:BSA)	Ref.
NENP-1 nanosheets	29	10	1:800	(S10)
MagG@COF-5	16	0.5	1:600	(S11)
mCTpBD	26	0.5	1:1000	(S12)
HFH-COFs@Au@GSH	25	0.1	1:1000	(S13)
$MnFe_2O_4$@C@APBA	23	0.5	1:800	(S14)
NUS-9	27	0.01	1:1500	(S15)
SCOF-2	28	0.01	1:5000	This work

Table S4. Detailed information of identified glycopeptides from healthy controls (group CK) and ovarian cancer patients (group HK) by SCOF-2. (A separate Excel file)

Table S5. Detailed information of identified glycoproteins from healthy controls (group CK) and ovarian cancer patients (group HK) by SCOF-2. (A separate Excel file)

Table S6. Detailed information of identified glycosylation sites from healthy controls (group CK) and ovarian cancer patients (group HK) by SCOF-2. (A separate Excel file)

Section 5. Supporting Reference

(S1) VandeVondele, J.; Krack, M.; Mohamed, F.; Parrinello, M.; Chassaing, T.; Hutter, J. *Comput. Phys. Commun.* **2005**, *167*, 103-128.

(S2) Krack, M.; Parrinello, M. *Phys. Chem. Chem. Phys.* **2000**, *2*, 2105-2112.

(S3) VandeVondele, J.; Hutter, J. *J. Chem. Phys.* **2007**, *127*, 114105.

(S4) Grimme, S.; Antony, J.; Ehrlich, S.; Krieg, H. *J. Chem. Phys.* **2010**, *132*, 154104.

(S5) Boersema, P. J.; Raijmakers, R.; Lemeer, S.; Mohammed, S.; Heck, A. J. R. *Nat. Protoc.* **2009**, *4*, 484-494.

(S6) Xu, J.; An, S.; Song, X.; Cao, Y.; Wang, N.; Qiu, X.; Zhang, Y.; Chen, J.; Duan, X.; Huang, J.; Li, W.; Wang, Y. *Adv. Mater.* **2021**, *33*, 2105178.

(S7) Wang, H.; Wang, T.; Ma, R.; Wu, K.; Li, H.; Feng, B.; Li, C.; Shen, Y. *J. Taiwan Inst. Chem. Eng.* **2020**, *112*, 122-129.

(S8) Wang, X.; Shi, B.; Yang, H.; Guan, J.; Liang, X.; Fan, C.; You, X.; Wang, Y.; Zhang, Z.; Wu, H.; Cheng, T.; Zhang, R.; Jiang, Z. *Nat. Commun.* **2022**, *13*, 1020.

(S9) Zheng, W.; Li, A.; Wang, X.; Li, Z.; Zhao, B.; Wang, L.; Kan, W.; Sun, L.; Qi, X. *New J.*

Chem. **2022**, *46*, 22185-22194.
(S10) Yang, S.-S.; Jiang, Y.-H.; Zhang, X.; Liu, L.-H.; Liu, S.; Zhang, H. *Anal. Methods* **2024**, *16*, 5304-5310.
(S11) Wang, J.; Li, J.; Gao, M.; Zhang, X. *Nanoscale* **2017**, *9*, 10750-10756.
(S12) Wu, Y.; Sun, N.; Deng, C. *ACS Appl. Mater. Interfaces* **2020**, *12*, 9814-9823.
(S13) Ji, Y.; He, Y.; Chen, R.; Zhong, C.; Li, H.; Wu, Y.; Lin, Z. *J. Mater. Chem. B* **2022**, *10*, 6507-6513.
(S14) Zhang, C.; Jin, X.; Wang, L.; Jin, C.; Han, X.; Ma, W.; Li, X.; Teng, G. *ACS Appl. Mater. Interfaces* **2021**, *13*, 9714-9728.
(S15) Ji, Y.; Li, H.; Dong, J.; Lin, J.; Lin, Z. *J. Chromatogr. A* **2023**, *1699*, 464020.
